# Respiratory–Swallow Coordination in Healthy Adults During Drinking of Thin to Extremely Thick Liquids: A Research Note

**DOI:** 10.1044/2019_JSLHR-19-00163

**Published:** 2020-02-27

**Authors:** Teresa J. Valenzano, Brittany T. Guida, Melanie Peladeau-Pigeon, Catriona M. Steele

**Affiliations:** a Rehabilitation Sciences Institute, University of Toronto, Ontario, Canada; b Swallowing Rehabilitation Research Laboratory, KITE, Toronto Rehabilitation Institute, University Health Network, Ontario, Canada

## Abstract

**Purpose:**

Respiratory–swallow coordination is vital for airway protection, preventing aspiration, or penetration of foreign material into the airway. With the implementation of the International Dysphagia Diet Standardization Initiative definitions for different liquid consistencies used in dysphagia management, it is important to establish whether respiratory–swallow coordination patterns differ across these consistencies. This study aimed to evaluate respiratory behaviors during swallowing across the spectrum from thin to extremely thick liquids in healthy adults less than 60 years of age.

**Method:**

Thirty healthy adults, aged 21–55 years, each consumed 54 naturally sized cup sips or spoonfuls of liquid stimuli prepared in thin, slightly thick, mildly thick, moderately thick, and extremely thick consistencies. Half of the stimuli were prepared using barium and half with a lemon-flavored water. Concurrent respiratory and swallowing pressure signals were collected to evaluate the respiratory phase pattern and pause duration associated with the swallow.

**Results:**

An expiration–swallow–expiration pattern was the dominant respiratory phase pattern, observed in 92.7% of the trials, with no significant effect of consistency. Respiratory pause duration was found to be significantly shorter with barium stimuli (0.73 s) compared to nonbarium stimuli (0.78 s) (*p* < .001, Cohen's *d* = .2), with no notable effects based on the factors of sex or liquid consistency.

**Conclusions:**

In a convenience sample of healthy adults under the age of 60 years, consistent respiratory–swallow phasing and stable timing across the spectrum from thin to extremely thick liquids was observed. The data from this study can serve as preliminary reference data to which assessment information for individuals with dysphagia or respiratory challenges can be compared.

Central pattern generators, located in the brainstem, coordinate the respiratory and swallowing systems. Coordinated behaviors between these two systems work to prevent the aspiration of foreign material into the airway through the precisely timed activation of the muscles and structures responsible for laryngeal vestibule closure, and the cessation of airflow through the oropharyngeal tract during swallowing (referred to as a *respiratory pause* or *swallow apnea*). The most commonly observed respiratory phase pattern in healthy swallowing is the expiration–respiratory pause–expiration pattern, which will, henceforth, be referred to as the *expiration–expiration* (E–E) pattern (Boden et al., 2009; Martin-Harris et al., 2003, 2005; McFarland et al., 1994; Paydarfar et al., 1995; Perlman et al., 2005; Preiksaitis & Mills, 1996). Postswallow expiration is thought to be an airway-protective mechanism, preventing remaining pharyngeal residue or penetrated bolus in the laryngeal vestibule from travelling further into the airway and potentially ejecting material from the vestibule back into the hypopharynx (Brodsky et al., 2010; Klahn & Perlman, 1999; Martin-Harris et al., 2005). Conversely, swallows in which the respiratory pause is followed by inspiratory airflow, as opposed to expiratory, are thought to involve a greater risk of postswallow aspiration of pharyngeal residue (Brodsky et al., 2010; Hadjikoutis et al., 2000; Martin-Harris et al., 2008; Nishino & Hiraga, 1991; Preiksaitis & Mills, 1996).

The respiratory pause is an apnea-like cessation of airflow during the swallow that is typically reported to have a duration of approximately 750 ms (Plonk et al., 2011; Todd et al., 2012). However, both the duration and the timing of respiratory pause onset can vary as a function of bolus characteristics, such as volume, viscosity, and individual characteristics such as age and sex (Hiss et al., 2004; Plonk et al., 2011; Todd et al., 2012). Hiss et al. (2004) demonstrated an effect of age on timing of respiratory pause onset for thin liquid, thickened liquids, and pureed consistencies, with older adults showing earlier onset of the respiratory pause compared to their younger counterparts. In addition, they found that an increase in bolus volume was associated with earlier respiratory pause onset. These results are thought to reflect adaptability of the swallowing system to maintain airway patency during swallowing tasks across different liquid consistencies within the context of an aging system and varying task challenges (Hiss et al., 2004). In an earlier study, Hiss et al. (2001) observed significantly longer respiratory pause durations in women than men, with this difference increasing with advancing age. The authors noted that longer durations of upper esophageal sphincter opening were also observed in females; given that the larynx typically remains closed throughout the period of upper esophageal sphincter opening, it makes sense that this might result in longer respiratory pause duration. Plonk et al. (2011) described longer respiratory pause durations in healthy older women compared to younger women; in their discussion, they pointed out that prolonged respiratory pauses could either reflect a protective mechanism or, conversely a maladaptive strategy, seen in the context of healthy aging.

The role of bolus consistency and its effect on respiratory pause timing and duration is currently a matter of debate. Hiss et al. (2004) revealed variations in respiratory pause onset timing as a function of bolus viscosity, with earlier onset noted with thinner liquid consistencies. They explained this finding in relation to the flow rate of the consistencies trialled, citing that physiological events, including respiratory pause onset, need not occur as quickly for thicker consistencies compared to thinner consistencies. However, Perlman et al. (2000) found no differences in timing measures of respiration in swallowing as a function of viscosity in healthy young adults.

Diet texture modification is one of the most commonly used interventions to improve swallowing safety for individuals with dysphagia (Garcia et al., 2005). This practice is embedded in the idea that alteration of the texture or consistency of liquids and foods can facilitate safer swallowing (Clavé et al., 2006). For liquid consistencies, the general principle assumed is that slowing the speed of bolus passage through the pharynx will facilitate timelier airway protection and prevention of aspiration. The International Dysphagia Diet Standardization Initiative (IDDSI) has recently established standardized terminology and definitions for modified textures and consistencies used in dysphagia management (Cichero et al., 2017). Five levels of liquid consistency are included (thin, slightly thick, mildly thick, moderately thick, and extremely thick), covering the spectrum from the fastest to the slowest flow. As the implementation of the IDDSI framework becomes internationally accepted and enforced, an understanding of how swallowing and respiratory coordination is influenced by the different flow characteristics of each liquid level is needed.

In this study, we analyzed respiratory pause timing and respiratory–swallow phase patterns in healthy adults less than 60 years of age using barium and nonbarium liquids of five different consistencies as defined by the IDDSI framework. We hypothesized that liquid consistency would have no effect on respiratory pause duration or phase pattern. Additionally, we hypothesized the presence of barium might lead to shorter respiratory pause durations, as previously shown in female supertasters by Todd et al. (2012), but would show no effect on respiratory phase pattern. Finally, we hypothesized that there would be no differences in the frequency of atypical respiratory phase patterns (i.e., non E–E patterns) between male and female participants, but that a difference would be seen in respiratory pause duration, with women demonstrating longer respiratory pauses during the swallow than men, as previously reported in the literature.

## Method

Data were collected as part of a larger study exploring the physiology of healthy swallowing across the range from thin to extremely thick liquid consistencies. This study was approved by the institutional review board at the University Healthy Network, Toronto. Informed written consent was obtained prior to participation in the study. The larger study consisted of two data collection sessions: (a) a videofluoroscopic swallow study and (b) an in-lab session. The data for the study described in this research note were collected during the in-lab session, held at the Swallowing Rehabilitation Research Lab at KITE—Toronto Rehabilitation Institute—University Health Network.

### Participants

Forty healthy adults under the age of 60 years were recruited for the larger study. The upper age boundary was based on an interest in factors related to tongue strength in the larger study; previous literature has suggested that age-related reductions in tongue strength can be detected after the age of 60 years, with further reductions in those aged 70 years and older (Vanderwegen et al., 2012). Participants were excluded if they reported a history of swallowing impairment, motor speech difficulties, gastro-esophageal difficulties, chronic sinusitis, or a neurological disorder. In addition, participants were excluded if they reported use of medications with potential impact on swallowing, salivary flow, smell, or taste. Due to the fact that the larger study involved one session of radiographic data collection under videofluoroscopy, children under the age of 18 years, pregnant women, or individuals with occupational radiation exposure were also excluded. Of the 40 individuals enrolled in the larger study, 33 agreed to the supplementary collection of respiratory signals using a nasal cannula, which was required for the current analysis. Technical problems with nasal cannula signal registration resulted in data being unavailable for three participants. The final data set available for analysis comprised data from 12 men (*M*_age_ = 35.4 years, range: 21–55 years) and 18 women (*M*_age_ = 32.8 years, range: 25–54 years), a convenience sample collected from the larger study data set.

### Apparatus

Concurrent respiratory and swallowing pressure data were collected using the KayPENTAX Digital Swallowing Workstation (DSW). The respiratory measurements were obtained using a nasal cannula by the thermistor method at a capture rate of 250 Hz; the temperature of the airflow was used to determine directionality with warmer temperatures reflecting expiratory airflow and lower temperatures reflecting inspiratory airflow. Each nasal cannula was calibrated through the KayPENTAX DSW to the temperature in the testing room immediately prior to the study session beginning and was then placed within the nares of the participant. The protocol also involved the concurrent measurement of tongue-palate pressures using a three-sensor tongue bulb array placed with adhesive on the palatal surface. Data regarding measures of tongue pressure during swallowing have been reported in a separate manuscript (Steele et al., 2019).

### Procedure

**Figure 1.**
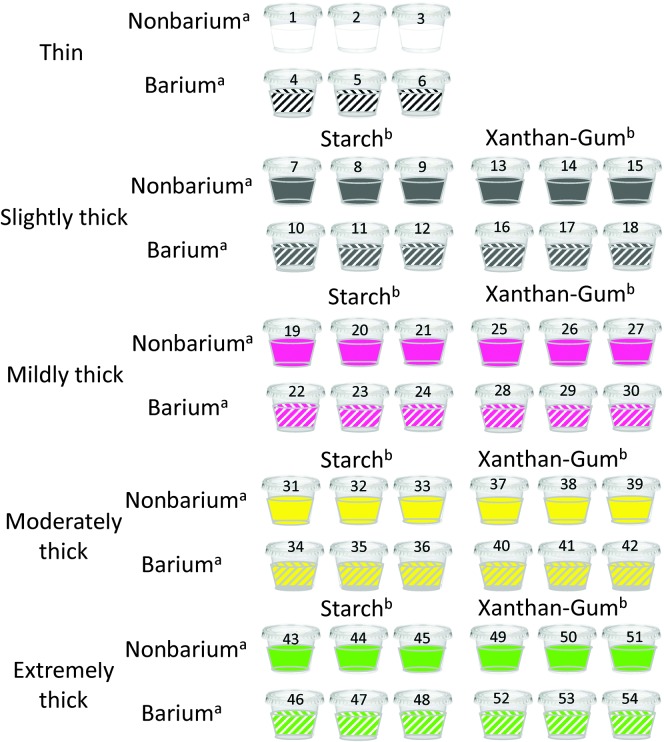
Study protocol. Nonbarium stimuli are indicated in solid colors. Barium stimuli are indicated in hashed colors. Numbers indicate the order of presentation. ^a^Randomization factor by presence of barium in stimuli. ^b^Randomization factor by thickener type.

Each participant was presented with 54 cups of liquid stimuli, organized in blocks of three cups. Each block consisted of the same stimulus in terms of consistency, type of thickening agent used (starch vs. gum), and presence or absence of barium. Where barium was included, we used Bracco E-Z-Paque powered barium, mixed with water in a 20% weight-to-volume concentration. The nonbarium stimuli were prepared using Nestlé Lemon Splash bottled water. In order to improve the palatability of the test stimuli, lemon-flavored water was used instead of unflavored thickened water. The intensity of the lemon flavor was rated as mild by a taste panel and was below concentration levels reported to influence swallowing behaviors (Nagy et al., 2014). The stimuli were presented in order of increasing thickness (thin, slightly thick, mildly thick, moderately thick, and extremely thick liquid), with randomized order of barium/nonbarium blocks and of thickener block (starch/gum) within barium condition. The two thickeners used were Nestlé Resource ThickenUp Clear, a gum-based thickener, and Nestlé Resource ThickenUp, a starch-based thickener, products that are commonly used in health care settings in North America, Europe, and Australasia. Each cup was filled with approximately 40 ml of liquid. Participants were instructed to take one natural sip from each cup, at their own pace. Sip volumes averaged 9.9 ml (95% confidence interval (CI) 8.5–11.1 ml]) for the thin nonbarium and 9.6 ml (95% CI [8.5–10.7 ml]) for the thin barium. Sip volumes were slightly smaller for the slightly and mildly thick stimuli (mean values ranging from 6.1 to 7.9 ml) and smaller again for the moderately and extremely thick stimuli, which were served by teaspoon (mean values ranging from 3.5 to 4.3 ml). After completing each block of stimuli, the participant took a sip of water to clear any oral or pharyngeal residue that might be present (see Figure 1).

During data collection, the recording was tagged in real time by a member of the research team to index the events of swallow onset (cup reaching the participant's lips) and swallow offset (observed return of the thyroid cartilage to its rest position). This allowed for the subsequent identification of segments associated with swallows in the nasal airflow signal.

### Data Postprocessing

**Figure 2.**
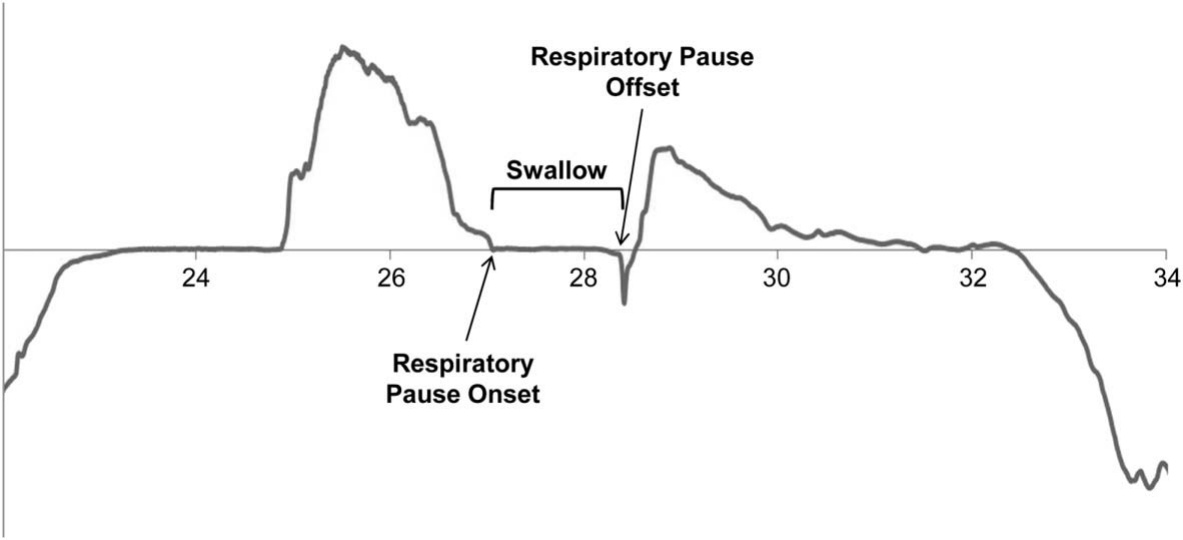
Respiratory pause duration.

Following completion of the data collection session, the nasal airflow waveforms were retrieved from the KayPENTAX DSW and segmented into bolus swallow portions using the tagged onset and offset events. Each segment was verified by two study team members through visual inspection of the waveforms to ensure a swallow was present. Two raters, previously trained to a high level of agreement, were each randomly assigned half of the data and independently analyzed half of the swallowing segments, identifying the respiratory phase pattern and respiratory pause duration for each swallow. Both raters were blinded to information about the trial number and bolus consistency. In the event of more than one swallow for a single bolus, only the initial swallow was used. Respiratory pause duration was measured from the onset of the respiratory pause, as indicated by a flat line on the nasal signal, to the offset of the respiratory pause and resumption of breathing (see Figure 2). Respiratory phase pattern was classified based on the presence of an inspiratory or expiratory pattern prior to and after the respiratory pause associated with the initial swallow. Four possible respiratory phase patterns could be identified: E–E, expiration–inspiration, inspiration–expiration, and inspiration–inspiration. Preswallow and postswallow respiratory phase patterns that were unable to be clearly identified were excluded from analysis. Preswallow airflow pattern data were available for 711 barium boluses and for 710 nonbarium boluses. Postswallow airflow pattern data were available for 752 barium boluses and for 745 nonbarium boluses. Combined pre- and postswallow data allowing identification of respiratory phase pattern was available for 710 barium boluses and for 704 nonbarium boluses.

### Analysis

Statistical analyses were performed in SPSS software (Version 25) using an α level of .05. A linear mixed-model analysis with repeated measures was conducted to identify differences in respiratory pause duration with factors of barium, consistency, and sex. This was followed by further explorations within barium condition for the thickened liquids only, with factors of consistency (i.e., slightly thick to extremely thick) and thickener type (starch, gum). For the investigations of respiratory phase pattern, frequencies for pre- and postswallow airflow direction (expiratory, inspiratory) and for the four possible pre- and postpatterns were tabulated overall by consistency. In order to explore possible consistency-based differences in the frequencies of atypical airflow patterns (i.e., preswallow inspiration, postswallow inspiration, and the resulting non E–E pattern), each participant's data were summarized at the consistency level overall, and within barium condition, using a binary scheme capturing the number of swallows for which an atypical (i.e., non–E–E) phasing pattern was seen (zero vs. one-or-more occurrences). Cochran's Q tests were performed to determine whether the frequency of atypical respiratory phase patterns differed across consistencies. Finally, in order to rule out potential sex differences in the frequency of atypical airflow patterns, two-by-two tables were developed, cross-tabulating participant sex by airflow pattern. For each parameter of interest (i.e., preswallow inspiration, postswallow inspiration, non–E–E respiratory phase pattern), four tables were drawn up (thin nonbarium, pooled thicker liquids nonbarium, thin barium, pooled thicker liquids barium), and Fisher's exact tests were performed to compare proportions of typical versus atypical respiratory phasing between the male and female participants.

## Results

### Respiratory Pause Duration

**Figure 3.**
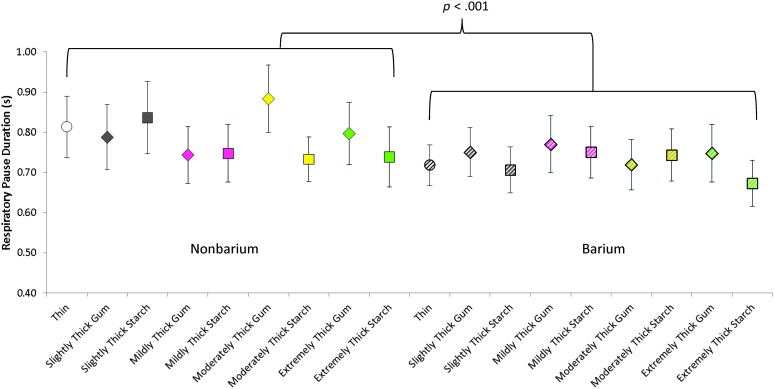
Duration of respiratory pause (s) by thickener type and liquid consistency.

Descriptive statistics for respiratory pause duration are reported in Table 1 as a function of barium and consistency. The mixed model found no significant effects of consistency, sex, or a Consistency × Sex interaction. However, a significant main effect of barium was found, *F*(1, 1384.14) = 12.243, *p* < .001, with shorter respiratory pause durations for the barium stimuli compared to the nonbarium stimuli (Cohen's *d* = 0.2, i.e., small). The further examination of thickener differences for the slightly to extremely thick stimuli found no significant effects (*p* > .05) of consistency, thickener, or Consistency × Thickener interactions in either the barium or nonbarium liquid subsets. These results are illustrated in Figure 3.

**Table 1.** Respiratory pause duration for barium and nonbarium liquid stimuli.

Barium condition	Consistency	Thickener	*M* (s)	*SD*	95% confidence interval	
					Lower bound	Upper bound
Nonbarium	Thin	None	0.81	0.34	0.74	0.89
	Slightly thick	Gum	0.79	0.37	0.71	0.87
		Starch	0.84	0.40	0.75	0.93
	Mildly thick	Gum	0.74	0.32	0.67	0.81
		Starch	0.75	0.33	0.68	0.82
	Moderately thick	Gum	0.88	0.38	0.80	0.97
		Starch	0.73	0.24	0.68	0.79
	Extremely thick	Gum	0.80	0.35	0.72	0.87
		Starch	0.74	0.32	0.66	0.81
Grand mean nonbarium			0.78	0.34	0.76	0.81
Barium	Thin	None	0.72	0.23	0.67	0.77
	Slightly thick	Gum	0.75	0.26	0.69	0.81
		Starch	0.71	0.26	0.65	0.76
	Mildly thick	Gum	0.77	0.31	0.70	0.84
		Starch	0.75	0.28	0.69	0.81
	Moderately thick	Gum	0.72	0.28	0.66	0.78
		Starch	0.74	0.29	0.68	0.81
	Extremely thick	Gum	0.75	0.32	0.68	0.82
		Starch	0.67	0.25	0.62	0.73
Grand mean barium			0.73	0.28	0.71	0.75

### Respiratory Phase Pattern

Table 2 shows overall frequencies (by bolus) for the four possible respiratory phase patterns by consistency. The E–E respiratory phase pattern was the most commonly produced pattern, followed by the inspiration–expiration and expiration–inspiration patterns; only three instances of the inspiration–inspiration respiratory phase pattern were observed across all boluses. Table 3 shows participant-level frequency distributions for atypical (i.e., inspiratory) airflow patterns by consistency within barium condition. The Cochran's Q tests by IDDSI level failed to find any significant differences in the frequencies of atypical phasing patterns across consistencies: preswallow airflow Cochran's Q = 1.273, *df* = 4, *p* = .87; postswallow airflow Cochran's Q = 1.176, *df* = 4, *p* = .88; respiratory phase pattern Cochran's Q = 0.364, *df* = 4, *p* = .99. Similarly, when frequencies of atypical phasing patterns were examined across stimuli grouped by barium condition and IDDSI level, no significant differences were found: pre-swallow airflow Cochran's Q = 9.43, *df* = 9, *p* = .4; postswallow airflow Cochran's Q = 11.64, *df* = 9, *p* = .23; respiratory phase pattern Cochran's Q = 11.61, *df* = 9, *p* = .24. The Fisher's exact tests exploring the frequency of atypical airflow patterns between male and female participants within consistency (thin vs. thick) and barium condition found a single significant sex difference, in the form of a higher frequency of postswallow inspiration on nonbarium thickened stimuli in the male participants (66.7% vs. 33.3%, *p* = .024).

**Table 2.** Summary of respiratory phase pattern frequencies across all bolus trials.

Respiratory phase pattern	Overall frequency (boluses)	%
Expiration–expiration (E–E)	1301	92.7
Inspiration–expiration (I–E)	43	3.1
Expiration–inspiration (E–I)	57	4.1
Inspiration–inspiration(I–I)	3	0.2
Total	1404	100.0

**Table 3.** Participant-level frequency distributions for atypical airflow patterns by consistency level within barium condition.

Barium condition	Consistency	Preswallow inspiration		Postswallow inspiration		Non–E–E respiratory phase pattern	
		*N*	%	*N*	%	*N*	%
Nonbarium	Thin	3	10	3	10	6	21
	Slightly thick	5	17	5	17	10	33
	Mildly thick	2	7	3	10	5	17
	Moderately thick	2	7	5	17	6	21
	Extremely thick	3	10	6	21	8	28
Barium	Thin	7	24	1	3	8	28
	Slightly thick	4	13	5	17	9	30
	Mildly thick	2	7	4	13	6	20
	Moderately thick	2	7	5	17	5	17
	Extremely thick	2	7	5	18	6	21

*Note.* E–E = expiration–expiration.

## Discussion

The aim of this preliminary study was to evaluate the coordination between respiration and swallowing across different liquid consistencies in a convenience sample of healthy adults less than 60 years of age. We hypothesized that the healthy adults in our sample would demonstrate respiratory phase pattern stability across the different IDDSI levels of liquid consistency, with possible differences in respiratory pause duration between barium and nonbarium liquids. As hypothesized, we found respiratory stability for pause duration and phase pattern, with no significant differences by consistency.

We found a significant difference in respiratory pause duration between barium and nonbarium liquids, with shorter durations with the barium condition stimuli (*M* = .73 s, 95% CI [.71–.75 s]) than the nonbarium condition (*M* = .78 s, 95% CI [.76–.81 s]), but a small effect size. This finding is consistent with observations previously reported in female supertasters by Todd et al. (2012). Furthermore, the mean values and CIs seen for thin nonbarium and barium stimuli in our study are very similar to those reported by Todd et al. This is reassuring; however, it must be noted that stimulus volume was fixed at 5 ml in the Todd et al. study, whereas the natural sips taken by participants were larger, with 95% CIs spanning 8.5–11.1 ml for the thin liquids, slightly smaller volumes for the slightly and mildly thick stimuli (mean values ranging from 6.1 to 7.9 ml) and smaller volumes again for the teaspoon-administered moderately and extremely thick stimuli (mean values ranging from 3.5 to 4.3 ml). Despite variations in sip volume, we found respiratory pause duration to be stable within the barium condition across all five liquid consistencies. This result is consistent with most of the reported literature, which has suggested stability in this measure across consistencies (Hiss et al., 2004). Similarly, the E–E respiratory phase pattern was the dominant pattern associated with the swallow, across both barium and nonbarium liquids and across all consistencies and in both sex groups. This finding is consistent with prior literature demonstrating stability in respiratory phase patterns across different liquid stimuli.

The nonbarium stimuli used in the study had a mild lemon flavoring, as judged by a blinded taste panel (Steele et al., 2019). Although the intensity of sourness was significantly lower than has been previously reported to impact swallowing behaviors (Nagy et al., 2014), the influence of taste cannot be ruled out. Furthermore, it must be noted that we did not conduct any measurement of genetic taste status in this study. Todd et al. (2012) conducted a study in healthy older versus younger women who were identified as supertasters or nontasters using a genetic taste status test. The supertaster group showed shorter respiratory pause durations with barium compared to water, suggesting that these individuals might be especially responsive to the influence of differences in stimulus taste (Todd et al., 2012). Interestingly in our study, although differences were seen in respiratory pause duration, no differences were seen in respiratory phase patterns across contrasting taste stimuli, with or without barium, with the E–E pattern dominating.

Evidence of stable respiratory pause durations and respiratory–swallow phase patterns in healthy control participants is important because it can serve as a reference for studies in patient groups with respiratory impairments that may influence the robustness of these coordinated systems and airway patency. For example, Troche et al. (2011) have found shorter respiratory pause durations for individuals with Parkinson's disease with an inspiratory pattern after the swallow. The duration of the respiratory pause may have implications for clinical swallowing assessments, including risk of penetration or aspiration of oral intake. Clinicians should consider the impact of respiratory stability during oral intake for those at risk of swallowing difficulty, particularly within the context of behavioral variability during drinking tasks, in factors such as sip volume and drinking rate/frequency, when the individual is eating in a naturalistic setting and not during a controlled assessment.

Methodological constraints that may have limited the results of the study include sample size, wide age range of the participants, and study design, particularly the fixed order of consistency in bolus presentation. Additionally, as swallowing events were confirmed visually and not concurrently with gold standard instrumentation, not all signals were able to be included in analysis, which may bias the data set toward “healthier” swallows. Reliability in signal identification was not completed for this study, although both raters had previously trained to a high level of agreement on a subset of the larger study data set. As this data set was composed of a convenience sample of the larger data set, not all of the included signals in this study were rated in duplication by the two blinded raters. As mentioned previously, this study was designed within the context of a larger research protocol, which called for each participant to also wear a three-sensor tongue bulb array placed with adhesive on the palatal surface. We cannot rule out the possibility that this could have caused a change in swallowing function or respiratory coordination.

## Conclusion

This study described the relationship between swallowing and respiration in healthy adults less than 60 years of age, establishing reference data for respiratory pause duration and respiratory phase pattern for the newly developed IDDSI liquid consistency levels. No difference was found in respiratory pause duration across the different liquid consistencies between the male and female participants. E–E was the most frequently occurring respiratory phase pattern, regardless of consistency and in both sexes. Further research should aim to expand these results by recruiting a larger sample size and comparing these data to an older population. In addition, future research should focus on duration and amplitude of the pre- and postswallow respiratory phase patterns to evaluate how liquid consistency may influence respiratory phase resetting in healthy adults.
